# Impact of the COVID-19 pandemic on domestic violence, 2019-2022^1^

**DOI:** 10.1590/1518-8345.7868.4744

**Published:** 2026-03-16

**Authors:** Teresa Mutemba Vilanculo, Leonora Rezende Pacheco, Karlla Antonieta Amorim Caetano, Rafael Alves Guimarães, Sheila Araújo Teles

**Affiliations:** 1 Universidade Federal de Goiás, Faculdade de Enfermagem, Goiânia, GO, Brazil.; 2 Scholarship holder at the Coordenação de Aperfeiçoamento de Pessoal de Nível Superior (CAPES), Brazil.

**Keywords:** Domestic Violence, Woman, Child, Elderly, Pandemics, COVID-19.

## Abstract

**Objective:**

to analyze the incidence and types of domestic violence against girls, adult women, and older adults before and during the COVID-19 pandemic.

**Method:**

ecological study. Overall incidence rates and rates by type of violence were calculated, and Poisson regression was performed to assess the impact of the COVID-19 pandemic on the rate of violence against girls, adult women, and older adults.

**Results:**

domestic violence was highly prevalent both before and after the pandemic, with an overall average rate of 235.32 cases per 100,000 population. However, a reduction was observed from 383.5 per 100,000 in the pre-pandemic period to 150.7 per 100,000 post-pandemic period. Life cycle data revealed that, despite the decline, sexual violence was most frequent among girls, and physical violence was most frequent among adult women. Among older women, abuse and financial abuse increased during the first year of the pandemic. Adult and older women had higher risks of violence - 6.35 and 3.7 times greater, respectively, compared to girls.

**Conclusion:**

domestic violence remains a serious public health concern. The reduction in cases during the pandemic likely reflects underreporting due to mobility restrictions.

## Highlights

**(1)** Violence against women is a significant public health issue in Maputo, Mozambique. **(2)** Adult women in Maputo are most vulnerable to domestic violence. **(3)** There was a decrease in reported cases of violence against women during the pandemic.

## Introduction 

Interpersonal violence is a complex and multi-causal public health phenomenon, responsible for high morbidity, including disability, hospitalizations, and mortality^1^ ^-^ ^2^. The impact of violence extends beyond the direct victims, also affecting the healthcare system and the economy, which face increasing strain due to the demand for health and social care^1^.

Interpersonal violence includes family or intimate partner violence, also known as domestic violence, which affects children, women, and the older women^1^. Although violence occurs in both sexes, women are disproportionately affected by interpersonal violence. Globally, an estimated 26% of women aged 15 and older have experienced physical and/or sexual violence by an intimate partner at some point in their lives, while the prevalence of violence, including non-intimate partners, reaches 30%^2^.

Violence against children includes all forms of violence perpetrated by parents, family members, caregivers, romantic partners, or any other person within their social circle or otherwise. A systematic review estimated that 54%, or more than one billion, of children aged 2 to 17 experienced some form of violence in the last 12 months^3^. Violence against the older women, also known as elder abuse, affects 15.7% of non-institutionalized older adults globally, with psychological and financial abuse being the main forms of violence^4^.

Considering the magnitude of violence as a public health problem, the United Nations (UN) included the elimination of violence against women and children in the Sustainable Development Goals (SDGs) of the 2030 Agenda. Furthermore, the World Health Organization (WHO) reinforces the role of the health system in policies aimed at combating violence through the Global Action Plan to strengthen the role of the health system within a multisectoral response to interpersonal violence, especially violence against women and children. The document also highlights the need for solid data to support effective public policies^5^.

In 2020, the COVID-19 pandemic imposed mobility restrictions and social distancing rules, resulting in increased domestic violence. However, the home may not be the safest place for people experiencing domestic violence, and the pandemic has contributed to an increase in cases of this type of violence^6^ ^-^ ^7^. In the United States, researchers showed that, immediately after the implementation of these measures, there was an increase in domestic violence, ranging from 10% in New York to 27% in Jefferson, Alabama^8^. In European countries, there was a 60% increase in emergency calls for domestic violence in 2020 compared to 2019^9^.

On the African continent, an estimated 33% of women have experienced some form of violence in their lifetime^2^. In Mozambique, domestic violence is a persistent issue, exacerbated by a patriarchal culture and the lack of resources for reporting and supporting victims^10^. Although the Mozambican government and non-governmental organizations have made significant efforts to strengthen legislation against domestic violence and improve the quality of life of survivors^11^, there is still little information about domestic violence in this country, particularly, about the impact of the pandemic on cases of violence. This information is essential to contribute to efforts to develop public policies to address this health problem, especially during periods of health crises, such as the COVID-19 pandemic.

The objective of this study was to analyze the incidence and types of domestic violence against girls, adult women, and older women in the city of Maputo, before and during the COVID-19 pandemic (2019-2022). 

## Method

### Study design

This is a retrospective ecological study, with a quantitative approach and descriptive and analytical components.

### Setting

The study was conducted in Maputo, the capital of Mozambique. According to the 2017 census, Mozambique has a population of 26,899,105 inhabitants and a Human Development Index (HDI) of 0.456, ranking ninth among the 189 countries assessed by the United Nations Development Program^12^ ^-^ ^13^. In Maputo, the estimated population is 1,118,387, of which 576,488 are women^12^.

### Period

The analysis covers reports from January 1, 2019, to December 31, 2022. Data were collected between January 2, 2023, and June 30, 2023.

### Population

The study population consisted of reported cases of domestic violence against girls, adult women, and older women residing in Maputo, Mozambique.

### Study variables

The following indicators were analyzed:

(i) Domestic violence rate: the indicator is calculated by the ratio between the absolute number of cases of violence against girls, adults, and older women and the corresponding resident population of Maputo, multiplied by 100,000.

(ii) Domestic violence rate by type: the indicator is calculated by the ratio between the absolute number of cases of violence against girls, adults, and older women by the corresponding resident population of Maputo, multiplied by 100,000.

This study analyzed criminal acts of violence against girls, adults, and older women, as described in Law No. 29/2009 of September 29, 2009, known as the Law on Domestic Violence Against Women, as follows: simple physical violence, severe physical violence, psychological violence, moral violence, non-consensual intercourse, intercourse with disease transmission, rape, and abuse.

The definitions of these types of violence is described in Law No. 29/2009^14^. For the purposes of this study, simple and severe physical violence were grouped and referred to as “physical violence”; psychological and moral violence were grouped and referred to as “psychological violence”; non-consensual intercourse, intercourse involving disease transmission, and rape were grouped and referred to as “sexual violence”; whereas property-related violence was referred to as “financial violence.”

### Data collection

Data were extracted from records of the Department of Assistance to Family and Minor Victims of Violence (DAFMVV) which compile data on reported cases of violence across the seven municipal districts of Maputo. In Mozambique, reports are made in person by the victim at police stations of the Republic of Mozambique (PRM) or the Office of Assistance to Family and Minor Victims of Violence (GAFMVV) in Maputo. In cases involving minors or individuals with physical or cognitive limitations, the legal guardian makes the report.

In addition to police station records, population projections for 2019, 2020, 2021, and 2022 were used, based on the last census conducted in the Republic of Mozambique in 2017, conducted by the National Institute of Statistics (INE).

The DAFMVV report presents age-grouped data, considering three life periods: girls (0-17 years old), adults (18-59 years old), and older women (≥ 60 years old).

### Data processing and analysis

The data were analyzed using the STATA statistical package, version 17.0. The absolute frequencies and rates of domestic violence overall and stratified by type of violence and age group were presented for the years 2019, 2020, 2021, and 2022.

The unit of analysis in this study includes reports of violence against girls, adults, and older women, aggregated according to the year of notification, the type of violence (physical, psychological, patrimonial, sexual, or abuse), the total number of reported cases, and the age group of the victims.

A Poisson regression model with robust variance was used to assess the impact of the COVID-19 pandemic on the rate of violence against women. The dependent variable of the model was the violence rate (per 100,000 women), and the independent variables included age group (0-17 years, 18-59 years, and >60 years) and a dummy variable, in which “0” represented the pre-pandemic period (from January 1, 2019, to December 31, 2019) and “1”, the pandemic period (from January 1, 2020, to December 31, 2022). The model results were expressed as Incidence Rate Ratio (IR) and respective 95% confidence intervals (95% CI). Statistical significance was assessed using the Wald test, adopting p < 0.05 as the significance threshold^15^.

### Ethical aspects

Secondary data from the public domain were used, obtained from the Department of Assistance to Family and Minors Victims of Violence, in Maputo City, Mozambique, and from the National Institute of Statistics. Because these are aggregated data that do not contain personally identifiable information about the study population, there was no need to obtain an Informed Consent Form. Therefore, the provisions of Resolution No. 510 of April 7, 2016, and Law No. 6/2023, the Human Health Research Law of the Republic of Mozambique, were complied with. 

## Results

Reported rates of domestic violence in Maputo decreased from 383.5 per 100,000 in 2019 to 150.7 between 2019 and 2022, as shown in Table 1 below. Physical violence was the most common, followed by psychological violence.

**Table 1:** Rates of domestic violence among girls, adults and older women, by typology, per 100,000 inhabitants in the city of Maputo, Mozambique, 2019-2022

Type of violence	Year			
	2019	2020	2021	2022
	n*/Rate	n*/Rate	n*/Rate	n*/Rate
Physical violence	1450/250.93	1160/200.46	763/131.64	383/65.96
Psychological violence	416/71.99	261/45.1	301/52.45	263/45.29
Financial violence	140/25.96	114/19.7	76/13.46	55/9.47
Sexual violence	167/28.9	136/23.5	113/19.5	97/16.7
Mistreatment	43/15.73	91/13.26	68/11.73	77/7.44
Total	2216/383.50	1762/304.49	1321/227.91	875/150.69

*n = Number of reported cases

Women were primarily victims of physical and psychological violence, with average rates of 271.69 and 89.28, respectively. Among girls, the highest average rate was sexual violence (44.60), followed by abuse (12.3). Meanwhile, among older women, the highest average rate was abuse (98.65), followed by physical (47.92), psychological (43.42), and financial (42.33) violence (Table 2).

**Table 2:** Overall average rate and by type of domestic violence among girls, adult women, and older women in Maputo City, Mozambique, 2019-2022

Type of violence	Girls (CI 95%)*	Adult women (CI 95%)*	Older women (CI 95%)*	All age groups (CI 95%)*
Physical violence	5.70 (-0.28-11.43)	271.69 (58.35-491.04)	47.92 (-36.72-132.55)	109.44 (17.28-201.59)
Psychological violence	0.91 (-0.11-1.94)	89.28 (59.98-118.59)	43.42 (-47.53-134.36)	44.54 (13.39-75.69)
Financial violence	0.34 (-0.36-1.05)	26.21 (9.47-42.94)	42.33 (-22.14-106.81)	22.96 (4.94-40.98)
Sexual violence	44.60 (34.21-54.98)	8.80 (-0.60-18.19)	6.32 (-3.39-16.03)	19.91 (7.77-32.04)
Mistreatment	12.03 (4.58-19.48)	4.77 (-6.2-15.76)	98.65 (-38.49-235.79)	38.48 (-1.85-78.81)
Total	63.59 (45.34-81.83)	403.75 (147.48-660.02)	238.64 (-16.97-494.25)	235.32 (16.13-354.52)

*CI 95% = 95% Confidence Interval

Table 3 presents the frequency of reported cases and the rate of domestic violence against girls, by type of violence, per 100,000 population, between 2019 and 2022. In 2019, 170 cases of violence against girls under 18 were reported, with a 20-percentage point reduction observed at the extremes of the period. Sexual violence was the most frequent, followed by abuse and physical violence. However, while sexual violence showed the same downward trend in the first year of the pandemic, with positive and negative fluctuations, there was an increase in the rate of abuse in 2020 (11.7 vs. 18.16).

**Table 3:** Frequency of reported cases and rate by type of domestic violence in girls under 18 years of age per 100,000 inhabitants in the city of Maputo, Mozambique, 2019-2022

Type of violence	Girls			
	2019	2020	2021	2022
	n* (rate)	n* (rate)	n* (rate)	n* (rate)
Physical violence	22 (9.90)	10 (4.57)	15 (6.94)	3 (1.40)
Psychological violence	3 (1.35)	2 (0.91)	3 (1.39)	0
Financial violence	1 (0.45)	0	2 (0.93)	0
Sexual violence	118 (53.10)	91 (41.57)	99 (45.81)	81 (37.9)
Mistreatment	26 (11.7)	41 (18.16)	18 (8.33)	20 (9.36)
Total	170 (76.5)	144 (65.78)	137 (63.40)	104 (48.66)

*n = Number of reported cases

Table 4 presents the frequency of reported cases and the rate of domestic violence among adult women, by type of violence, per 100,000 population. There was a decrease in the overall rate of violence over the period, from 594.19 in 2019 to 213.94 per 100,000 women in 2022. Physical violence was the most frequent type, followed by psychological and financial violence. Sexual violence rates remained stable in 2019 (14.23) and 2020 (13.52), before falling sharply in 2021 (2.98), with an increase in 2021 (4.45). Abuse among women was uncommon in 2019 and 2020, but in 2021, a rate of 14.92 per 100,000 women was observed. 

**Table 4:** Frequency of reported cases and rate by type of domestic violence in adult women per 100,000 inhabitants in the city of Maputo, Mozambique, 2019-2022

Type of violence	Adult women			
	2019	2020	2021	2022
	n* (rate)	n* (rate)	n* (rate)	n* (rate)
Physical violence	1398 (423.38)	1134 (340.64)	744 (221.98)	380 (112.77)
Psychological violence	381 (115.39)	249 (74.8)	298 (88.91)	263 (78.05)
Financial violence	133 (40.28)	90 (27.03)	74 (22.08)	52 (15.41)
Sexual violence	47 (14.23)	45 (13.52)	10 (2.98)	15 (4.45)
Mistreatment	3 (0.91)	0	50 (14.92)	11 (3.26)
Total	1962 (594.19)	1518 (455.99)	1176 (350.87)	721 (213.96)

*n = Number of reported cases

In 2019, the global rate of reported elder abuse was 369.71 per 100,000 older women, with a substantial decline in 2021 (45.81) and an increase in 2022 due to elder abuse (Table 5). Physical and psychological violence were the most frequent in 2019, but continued to decline throughout the pandemic, with no cases recorded in 2022. In contrast, the rate of financial abuse and, more sharply, elder abuse increased by approximately 40% in 2020. In 2021, the rate of financial abuse declined to 7.05, remaining at 10 cases with a slight increase in 2022. On the other hand, there were no reports of elder abuse in 2021, but in 2022 the rate reached 153.37 cases per 100,000 older women.

**Table 5:** Frequency of reported cases and rate by type of domestic violence among elderly women per 100,000 inhabitants in the city of Maputo, Mozambique, 2019-2022

Type of violence	Older women			
	2019	2020	2021	2022
	n* (rate)	n* (rate)	n* (rate)	n* (rate)
Physical violence	30 (117.99)	16 (59.57)	4 (14.09)	0
Psychological violence	32 (125.86)	10 (37.23)	3 (10.57)	0
Financial violence	16 (62.93)	24 (89.36)	2 (7.05)	3 (10.0)
Sexual violence	2 (7.87)	0	4 (14.09)	1 (3.33)
Mistreatment	14 (55.06)	50 (186.16)	0	46 (153.37)
Total	94 (369.71)	100 (372.33)	13 (45.81)	50 (166.71)

*n = Number of reported cases

The Poisson regression model with robust variance showed that during the pandemic period there was a 42% reduction (RTI: 0.58; 95% CI: 0.43-0.79) in the incidence rate of violence when compared to the pre-pandemic period, indicating a significant impact of the COVID-19 pandemic on the decrease in rates. Additionally, the analysis revealed that adult women (18-59 years) had a 535% higher incidence rate (RTI: 6.35; 95% CI: 4.78-8.43), while older women (>60 years) had a 270% higher rate (RTI: 3.70; 95% CI: 2.14-6.40), both compared to the 0-17 age group (children and adolescents).

## Discussion

This ecological study analyzed cases of domestic violence against girls, women, and older adults during the pre-pandemic (2019) and pandemic (2020-2022) periods in Maputo City, Mozambique. This approach was crucial for understanding changes in violence levels before and during the COVID-19 pandemic, as well as observing variations in types of violence and among different age groups. The analysis allowed us to identify important trends, such as the reduction in reported cases during the pandemic and the variation in the impact of different types of violence on different age groups, providing a comprehensive view of the effects of the public health crisis on domestic violence.

Estimates of violence against women on the African continent have shown high frequencies, and this study confirms this scenario in Maputo, the capital of Mozambique^2^. Even with the decrease in reports over the years studied, the number of cases of physical, psychological, sexual violence, and mistreatment is significant, which confirms an alarming reality.

In Mozambique, data from the 2011 Demographic and Health Survey^12^ revealed that 37% of women aged 15 and over had been victims of physical and/or sexual violence throughout their lives, with almost all cases perpetrated by their partners. The local culture naturalizes violence against women, and this certainly contributes to the scenario presented in this study. According to the same document, 23% of women gave at least one reason for their husband to abuse his wife, and 19% of men gave at least one reason that would justify their husband’s abuse of his wife^12^.

In the context of the pandemic, the quarantine measures adopted to contain COVID-19, while essential for protecting public health, had unexpected consequences, such as a significant increase in domestic violence, which disproportionately affected women^6^. Social isolation, necessary to reduce contagion, created a paradox: while people were physically isolated to protect themselves, many became more vulnerable within their own homes^7^. 

For some authors, the lockdown gave perpetrators more freedom, increasing cases of violence in several countries, such as Australia, China, various states in the United States, and even the United Kingdom^16^. Domestic isolation prevented many victims from accessing help, increasing their vulnerability.

However, this study shows that between 2019 and 2022, there was an approximately 60% reduction in the number of reported cases of violence against women in the city of Maputo. Physical violence was the most common, followed by psychological and sexual violence. Analysis by age group showed that adult and older women faced primarily physical and psychological violence, while girls were more likely to be victims of sexual violence. In the case of elderly women, there was an increase in abuse, especially in 2022. Furthermore, Poisson regression analysis indicated that adult and elderly women (RPA: 6.35 and RPA: 3.7) have a higher risk of suffering violence than girls and that the incidence of violence was lower in the pandemic period compared to the pre-pandemic period. 

Although there was a decrease, the number of domestic violence cases was significant in the year before the pandemic, demonstrating that violence against women is a significant problem in the city, especially among adult women, and that the decline in cases was most likely due to underreporting. Factors such as fear of reporting the violence, combined with fear of retaliation from family and society, and financial dependence linked to low educational attainment and limited income, lead many women to refrain from formalizing their complaints^17^. These factors create a context in which barriers to reporting become stronger, exacerbating the underreporting of violence cases.

The failure to report cases of violence is a significant obstacle in addressing this problem. By preventing violence from being properly recorded and visible, it hinders the development of public prevention policies and the provision of support services to victims. This situation undermines the ability of authorities and institutions to act effectively to break the cycle of violence and provide the necessary protection^18^.

A study conducted in Maputo^19^, also in the pre-pandemic period (April 2019) and prior to the Declaration of a State of Emergency (March 2020) and trans-pandemic (May to June 2020), revealed an increasing trend in the number of cases of violence, with 83 cases reported in April 2019 and 169 cases in March 2020.

However, as shown in this study, in May, the number of cases declined to 131 reported cases. According to this study, the apparent reduction should not reflect reality, since, in Mozambique, reports of violence at PRM and GAFMVV are made in person, confirming the suspicion of underreporting. 

Thus, the reduction in reports over the period under investigation may be less a reflection of a true decrease in violence and more a consequence of factors such as fear of reporting, financial dependence, or reduced access to support services. This suggests that, although the numbers have decreased, violence remained high.

Sexual violence was common among girls under 18. This age range is very broad, and the data did not allow us to disaggregate it into younger age groups to identify which age ranges are most prevalent in these types of violence. It is hypothesized that sexual violence may have occurred more frequently among adolescents, and that abuse may be more prevalent among children still dependent on the care of parents or guardians.

Abuse was more common among girls and older women. Restricted mobility, social isolation, and increased family tensions can make children more irritable, leading to aggressive and disobedient behavior, thus increasing the risk of domestic violence^20^. In the case of older women, greater dependence on others to perform basic tasks, limited social support due to isolation, and insufficient reporting mechanisms^21^ are factors that may have led to underreporting and fluctuations in violence cases among these age groups in the city of Maputo.

Among older women, there was also an increase in financial violence in the first year of the pandemic, with a subsequent decline. The mobility limitations imposed by the COVID-19 pandemic impacted the global economy and, consequently, the household economy^22^. Therefore, older people may have suffered abuse by relying on their assets, pensions, and retirement benefits to support their families. 

The reduction in the incidence rate of violence in Maputo city, observed in the Poisson regression analysis, indicates possible underreporting during the pandemic period and not necessarily a true protective effect on the incidence of domestic violence during the pandemic period (2020-2022) compared to the pre-pandemic period (2019). 

While at first glance this reduction may appear positive, interpreting this effect is complex and may reflect several contextual and structural factors^23^. The true extent of violence may be masked by several factors beyond underreporting, such as changes in the prevalent types of violence and restrictions on access to support and protection services, suggesting the need to improve reporting systems and ensure that victims have access to necessary resources, regardless of external conditions, such as the pandemic.

The results on domestic violence against women throughout their life cycles in Maputo city between 2019 and 2022 can be applied in practice to guide various interventions and changes in the areas of health, social assistance, and public policy.

Some practical actions can be implemented to address the problem of domestic violence^5^, such as:

- Strengthening health and reporting services to improve identification, care, and reporting, including training health professionals to identify signs of violence, even if victims do not report it directly; establishing systematic screening protocols in all health appointments, including gynecological, pediatric, and geriatric appointments; and improving data recording and reporting to ensure that all identified cases are recorded and reported to the authorities;
- Developing integrated support networks that offer comprehensive assistance to women experiencing violence, creating shelter centers where people experiencing violence can seek immediate help and obtain legal, psychological, and medical support, which are accessible and operate 24 hours a day;
- Community awareness and education programs, implementing actions such as: educational programs to educate communities about the different types of violence (physical, psychological, financial, abuse, and sexual) and how to report it, through media campaigns, lectures in schools and community centers, and radio programs; encouraging the empowerment of women, especially the most vulnerable, through education, professional training, and financial support programs, as women with greater independence are better able to report aggressors and leave abusive relationships;
- Implementation of remote support services to ensure that women experiencing violence receive support even when mobility is restricted, providing free emergency phone numbers or apps that allow anonymous reporting; and websites or apps where victims can access information on how to seek help, in addition to offering emotional support through chats with mental health professionals;
- Interventions targeted at specific age groups to meet the needs of each group, focusing on programs to prevent sexual violence and abuse, equipping schools with education programs on rights, self-care, and how to report abuse; offering programs that address physical and psychological violence, focusing on resilience strategies, emotional and economic support; and developing specific interventions to combat abuse and abuse of older women, including awareness campaigns on financial abuse and neglect and the creation of specialized support services;
- Continuous monitoring and evidence-based policies: Local and national governments should use data from violence reporting and research to monitor trends and develop evidence-based public policies that address the realities of women in different contexts;
- Ongoing training for health and social care professionals who work directly with victims, with periodic training on best practices for dealing with violence, reception techniques, and legal procedures essential to improving the response of health and social services; and training programs that emphasize the need to treat violence cases sensitively and adapt to different cultural, economic, and social contexts.

Based on the results presented, it is suggested that future research address topics such as:

- Barriers to reporting: investigate how isolation measures during the pandemic affected women’s ability to report violence, considering factors such as lack of access to electronic devices and low familiarity with technology among vulnerable populations; and assess how to expand access to and the effectiveness of alternative reporting channels, such as online platforms or telephone services.
- Violence against older women and changing patterns of violence: Investigate the causes behind the increase in cases of elder abuse and the decrease in cases of physical and psychological violence; and understand the factors that lead to financial vulnerability and abuse.
- Violence against girls: Analyze in more detail the increase in abuse and the variation in cases of sexual violence against girls under 18, identifying the most effective preventive and protective measures for this age group.
- Social and cultural determinants of violence: Investigate the social, cultural, and economic factors that contribute to violence against women in the city of Maputo; and study how poverty, unemployment, gender norms, and family structure influence the dynamics of violence, in addition to exploring how these variables affect women across different life cycles.

Limitations of the study include aspects related to the quality of records in Maputo, which varied over the four years studied, hindering a more precise analysis. Additionally, the data provided were aggregated from the seven municipal districts of Maputo City, which prevented a territorial analysis of the incidents. The lack of information on the demographic profiles of victims and perpetrators limited understanding of the dynamics of the cases. Finally, variations in the way data were aggregated by age group made direct comparisons between different age groups difficult.

## Conclusion

Analysis of violence against women in Maputo between 2019 and 2022 reveals important patterns and context-specific trends in terms of age, temporal, and contextual variations, such as the pandemic. Adult women remain the most affected by violence over the years, reflecting their high exposure to different forms of violence, such as physical, psychological, and financial. Older women also experienced a significant rate of violence, being more likely to suffer abuse than children, with a notable increase in abuse between 2020 and 2022. During the pandemic, there was a 42% reduction in reported cases of violence compared to the pre-pandemic period. This decrease may be associated with social confinement, changes in daily routines, and reduced reporting capacity, in addition to the socioeconomic impact caused by the pandemic, which altered family and social dynamics, making it difficult to identify and report cases of violence. Therefore, this reduction should be analyzed with caution, as it may not reflect a true decrease in violence, but rather a reduction in reported cases due to restrictions and limited access to protective services.

Even though there was a reduction in cases during the pandemic, it is essential to monitor the post-pandemic scenario to understand whether the downward trend continues or whether new factors emerge that could increase violence. Training health, education, and social services professionals to identify and intervene in cases of violence is crucial, as is raising public awareness of the issue. Continued data collection and monitoring of cases of violence is an important strategy for identifying emerging trends in effectively combating violence.

In short, although the data indicate a downward trend in violence against women in Maputo City, challenges remain, especially regarding abuse and sexual violence against girls. Strengthening support networks and promoting prevention policies, along with consolidating surveillance and social protection systems, are essential to address this serious social problem and ensure the safety and well-being of the female population, promoting a safer and more welcoming environment.

## Data Availability

Datasets related to this article will be available upon request to the **corresponding author.**
