# Supplementary material for: Impact of the COVID-19 pandemic on domestic violence, 2019-2022
Source: Rev Lat Am Enfermagem. 2026 Mar 16;34:e4744. doi: 10.1590/1518-8345.7868.4744 (PMC12991390; doi:10.1590/1518-8345.7868.4744)
Supplement: Translation

# Translation: Impacto da pandemia da COVID-19 na violência doméstica, 2019-2022^2^

**DOI:** 10.1590/1518-8345.7868.4745

**Authors:** Teresa Mutemba Vilanculo, Leonora Rezende Pacheco, Karlla Antonieta Amorim Caetano, Rafael Alves Guimarães, Sheila Araújo Teles

**Affiliations:** 1 Universidade Federal de Goiás, Faculdade de Enfermagem, Goiânia, GO, Brasil.; 2 Bolsista da Coordenação de Aperfeiçoamento de Pessoal de Nível Superior (CAPES), Brasil.

**Keywords:** Violência Doméstica, Mulher, Criança, Idoso, Pandemias, COVID-19.

## Abstract

**Objetivo:**

analisar a incidência e os tipos de violência doméstica contra meninas, mulheres adultas e idosas, antes e durante a pandemia de COVID-19.

**Método:**

estudo ecológico. Foram calculadas taxas de incidência global e por tipo de violência e foi realizada regressão de Poisson para avaliar o impacto da pandemia de COVID-19 sobre a taxa de violência contra meninas, mulheres adultas e idosas.

**Resultados:**

verificou-se uma frequência elevada de violência doméstica no período pré e trans pandemia, com taxa média geral de 235,32 casos/100.000 habitantes. Contudo, observou-se redução de 383,5/100.000 (período pré-pandemia) para 150,7/100.000 (período trans pandemia). Os dados por ciclo de vida mostraram que, apesar do declínio, a violência sexual e a violência física foram as mais frequentes em meninas e mulheres, respectivamente. Em idosas, maus tratos e violência financeira aumentaram no primeiro ano da pandemia. Mulheres adultas e idosas apresentaram maior risco de violência, 6,35 e 3,7 vezes em comparação às meninas.

**Conclusão:**

a violência doméstica é um grave problema de saúde pública. A redução de casos durante a pandemia sugere subnotificação devido às restrições impostas pela pandemia.

## Destaques

**(1)** Violência contra mulher é um problema de saúde pública em Maputo, Moçambique. **(2)** Mulheres adultas em Maputo são as mais vulneráveis à violência doméstica. **(3)** Houve redução de notificação de casos de violência contra mulheres durante a pandemia.

## Introdução 

A violência interpessoal é um fenômeno complexo e multicausal de saúde pública, sendo responsável por alta morbidade, incluindo incapacidades, hospitalizações e mortalidade^1^ ^-^ ^2^. O impacto da violência vai além das vítimas diretas, afetando também o sistema de saúde e economia, que enfrenta sobrecarga crescente devido à demanda por assistência em saúde e social^1^.

A violência interpessoal inclui a violência familiar ou por parceiro íntimo, também denominada violência doméstica que afeta crianças, mulheres e idosos^1^. Embora a violência ocorra em ambos os sexos, as mulheres são desproporcionalmente afetadas por violência interpessoal. Globalmente, estima-se que 26% das mulheres com 15 anos ou mais de idade já sofreram violência física e/ou sexual por parceiro íntimo em algum momento da vida, enquanto a prevalência de violência, incluindo parceiros não íntimos, alcança 30%^2^. 

A violência contra criança inclui todas as formas de violência perpetradas por pais, familiares, cuidadores, parceria romântica, ou qualquer outra pessoa do seu círculo social ou não. Uma revisão sistemática estimou que 54%, ou seja, mais de um bilhão de crianças de 2 a 17 anos sofreram alguma forma de violência nos últimos 12 meses^3^. Já a violência contra idosos, também denominada abuso de idosos, acomete 15,7% dos idosos não institucionalizados globalmente, sendo o abuso psicológico e financeiro as principais formas de violência^4^.

Considerando a magnitude da violência como problema de saúde pública, a Organização das Nações Unidas (ONU) incluiu a eliminação da violência contra mulheres e crianças nos Objetivos de Desenvolvimento Sustentável (ODS) da Agenda 2030. Além disso, a Organização Mundial de Saúde (OMS) reforça o papel do sistema de saúde em políticas destinadas ao enfrentamento da violência, por meio do *Plano de Ação Global para fortalecer o papel do sistema de saúde dentro de uma resposta multisetorial dirigida à violência interpessoal, em especial a violência contra mulheres e crianças*. O documento destaca, ainda, a necessidade de dados sólidos para embasar políticas públicas eficazes^5^.

Em 2020, a pandemia de COVID-19 impôs restrições de mobilidade e regras de distanciamento social que resultaram em maior convivência doméstica. Contudo, o lar pode não ser o local mais seguro para pessoas que sofrem violência doméstica e o cenário pandêmico contribuiu para o aumento de casos de desse tipo de violência^6^ ^-^ ^7^. Nos Estados Unidos da América^8^, pesquisadores mostraram que, logo após a implementação dessas medidas, houve um aumento de violência doméstica, variando de 10% em Nova Iorque, a 27% em Jefferson, Alabama. Em países europeus, houve um aumento de 60% de chamadas de emergência por violência doméstica em 2020 comparado a 2019^9^.

No continente africano, estima-se que 33% das mulheres já sofreram alguma forma de violência na vida^2^. Em Moçambique, a violência doméstica é uma questão persistente, agravada pela cultura patriarcal e pela escassez de recursos para a denúncia e apoio às vítimas^10^. Embora o governo moçambicano e entidades não governamentais tenham feito um grande esforço para fortalecer a legislação contra violência doméstica e melhorar a qualidade de vida dos sobreviventes^11^, ainda são poucas as informações sobre violência doméstica nesse país, e, em particular, sobre o impacto da pandemia nos casos de violência. Essas informações são fundamentais para contribuir com os esforços para a construção de políticas públicas de enfrentamento a esse agravo à saúde, principalmente em períodos de crises sanitárias, como a da pandemia da COVID-19.

O objetivo do presente estudo foi analisar a incidência e os tipos de violência doméstica contra meninas, mulheres adultas e idosas na cidade de Maputo, antes e durante a pandemia de COVID-19 (2019-2022). 

## Método

### Delineamento do estudo

Trata-se de um estudo ecológico retrospectivo, de abordagem quantitativa, com componentes descritivos e analíticos.

### Local 

O estudo foi conduzido na cidade de Maputo, capital de Moçambique. Segundo o censo demográfico de 2017, Moçambique possui uma população de 26.899.105 habitantes e um Índice de Desenvolvimento Humano (IDH) de 0,456, posicionando-se como o nono pior entre os 189 países avaliados pelo Programa das Nações Unidas para o Desenvolvimento^12^ ^-^ ^13^. Em Maputo, a população estimada é de 1.118.387, sendo 576.488 mulheres^12^.

### Período

O período de seleção (recorte) da demanda analisada foi de 01 de janeiro de 2019 a 31 de dezembro de 2022. A coleta de dados foi realizada de 02 de janeiro a 30 de junho de 2023.

### População 

A população do estudo foi composta pelos casos notificados de violência doméstica contra meninas, mulheres adultas e idosas residentes na cidade de Maputo, Moçambique. 

### Variáveis do estudo

Foram analisados os seguintes indicadores:

(i) Taxa de violência doméstica: o indicador é calculado pela razão entre o número absoluto de casos de violência em meninas, adultas e idosas e a população residente correspondente de Maputo, multiplicado por 100.000. 

(ii) Taxa de violência doméstica por tipologia: o indicador é calculado pela razão entre o número absoluto de casos de violência por tipologia em meninas, adultas e idosas e a população residente correspondente de Maputo, multiplicado por 100.000. 

Neste trabalho, foram analisados os atos criminais de violência contra meninas, adultas e idosas, descritas na Lei nº 29/2009 de 29 de setembro de 2009, denominada como Lei da Violência Doméstica Praticada contra Mulher, conforme a seguir: violência física simples, violência física grave, violência psicológica, violência moral, copula não consentida, cópula com transmissão de doença, violação, maus tratos. 

A definição desses tipos de violência é descrita na Lei nº 29/2009^14^. Para fins deste estudo, violência física simples e grave foram agrupadas e denominadas como “violência física”; já violência psicológica e moral foram agrupadas e denominadas como “violência psicológica”; por sua vez, cópula não consentida, cópula com transmissão de doença e violação foram agrupadas e denominadas como “violência sexual”; enquanto violência patrimonial foi denominada “violência financeira”. 

### Coleta de dados

Os dados foram extraídos de registros do Departamento de Atendimento à Família e Menores Vítimas de Violência (DAFMVV), que compilam os dados de notificações de violências nos sete distritos municipais da cidade de Maputo. Em Moçambique, as notificações são realizadas presencialmente pela vítima nas delegacias da Polícia da República de Moçambique (PRM) ou Gabinete de Atendimento à Família e Menores Vítimas de Violência (GAFMVV) de Maputo. Em casos de menores de idade ou pessoas com limitações físicas ou cognitivas, o responsável legal realiza a notificação.

Além dos registros das delegacias, foram utilizadas as projeções populacionais para 2019, 2020, 2021 e 2022, a partir do último censo realizado na República de Moçambique em 2017, conduzido pelo Instituto Nacional de Estatística (INE). 

O relatório da DAFMVV apresenta dados agrupados de idade, considerando três períodos de vida: meninas (0-17 anos), adultas (18-59 anos) e idosas (≥ 60 anos).

### Tratamento e análise de dados

Os dados foram analisados utilizando o pacote estatístico STATA, versão 17.0. Foram apresentadas as frequências absolutas e as taxas de violência doméstica global e estratificadas por tipo de violência e faixa etária para os anos de 2019, 2020, 2021 e 2022.

A unidade de análise neste estudo inclui as notificações de violência contra meninas, adultas e idosas que foram agregadas de acordo com o ano de notificação, a tipologia da violência (física, psicológica, patrimonial, sexual e maus tratos), o número total de casos registrados e a faixa etária das vítimas. 

Um modelo de regressão de Poisson com variância robusta foi utilizado para avaliar o impacto da pandemia de COVID-19 sobre a taxa de violência contra a mulher. A variável dependente do modelo foi a taxa de violência (por 100.000 mulheres), e as variáveis independentes incluíram a faixa etária (0-17 anos, 18-59 anos e >60 anos) e uma variável *dummy*, em que “0” representou o período pré-pandêmico (de 01/01/2019 a 31/12/2019) e “1”, o período pandêmico (de 01/01/2020 a 31/12/2022). Os resultados do modelo foram expressos como Razão de Taxa de Incidência (RTI) e respectivos intervalos de confiança de 95% (IC95%). A significância estatística foi avaliada por meio do teste de Wald, adotando-se p < 0,05 como limiar de significância^15^.

### Aspectos éticos

Utilizou-se dados secundários de domínio público, obtidos junto ao Departamento de Atendimento à Família e Menores Vítimas de Violência, na Cidade de Maputo, Moçambique, e do Instituto Nacional de Estatística. Por se tratar de dados agregados que não contêm informações pessoais identificáveis da população em estudo, não houve necessidade de obtenção de um Termo de Consentimento Livre e Esclarecido (TCLE). Assim, cumpriu-se as determinações da Resolução nº 510, de 7 de abril de 2016 e a Lei nº 6/2023, Lei de Investigação em Saúde Humana da República de Moçambique.

## Resultados

A Tabela 1 apresenta as taxas de violência doméstica por 100.000 habitantes na Cidade de Maputo, Moçambique, entre 2019 e 2022. As taxas totais diminuíram ao longo dos quatro anos, de 383,50 por 100.000 habitantes em 2019 para 150,7 em 2022, refletindo uma queda acentuada nas taxas de violência reportadas. A violência física foi o tipo de violência mais frequente, seguida da violência psicológica. 

**Tabela 1:** Taxas de violência doméstica em meninas, adultas e idosas, por tipologia, por 100.000 habitantes na cidade de Maputo, Moçambique, 2019-2022

Tipo de violência	Ano			
	2019	2020	2021	2022
	n*/Taxa	n*/Taxa	n*/Taxa	n*/Taxa
Violência física	1450/250,93	1160/200,46	763/131,64	383/65,96
Violência psicológica	416/71,99	261/45,1	301/52,45	263/45,29
Violência financeira	140/25,96	114/19,7	76/13,46	55/9,47
Violência sexual	167/28,9	136/23,5	113/19,5	97/16,7
Maus tratos	43/15,73	91/13,26	68/11,73	77/7,44
Total	2216/383,50	1762/304,49	1321/227,91	875/150,69

*n = Número de casos notificados

Mulheres foram vítimas principalmente de violências física e psicológica, com taxas médias de 271,69 e 89,28, respectivamente. Já em meninas, a maior taxa média foi de violência sexual (44,60), seguida de maus tratos (12,3). Por outro lado, em idosas, a maior taxa média foi a incidência de maus tratos (98,65), seguida de violência física (47,92), psicológica (43,42) e financeira (42,33) (Tabela 2).

**Tabela 2:** Taxa média global e por tipo de violência doméstica em meninas, mulheres adultas e idosas na Cidade de Maputo, Moçambique, 2019-2022

Tipo de violência	Meninas (IC95%)*	Adultas (IC95%)*	Idosas (IC 95%)*	Todas as faixas etárias (IC95%)*
Física	5,70 (-0,28-11,43)	271,69 (58,35-491,04)	47,92 (-36,72-132,55)	109,44 (17,28-201,59)
Psicológica	0,91 (-0,11-1,94)	89,28 (59,98-118,59)	43,42 (-47,53-134,36)	44,54 (13,39-75,69)
Financeira	0,34 (-0,36-1,05)	26,21 (9,47-42,94)	42,33 (-22,14-106,81)	22,96 (4,94-40,98)
Sexual	44,60 (34,21-54,98)	8,80 (-0,60-18,19)	6,32 (-3,39-16,03)	19,91 (7,77-32,04)
Maus tratos	12,03 (4,58-19,48)	4,77 (-6,2-15,76)	98,65 (-38,49-235,79)	38,48 (-1,85-78,81)
Total	63,59 (45,34-81,83)	403,75 (147,48-660,02)	238,64 (-16,97-494,25)	235,32 (16,13-354,52)

*IC95% = Intervalo de Confiança de 95%

A Tabela 3 apresenta a frequência de casos notificados e a taxa de violência doméstica em meninas, por tipo de violência, por 100.000 habitantes, entre 2019 e 2022. Em 2019 foram notificados 170 casos de violência contra meninas menores de 18 anos, sendo observada uma redução de 20 pontos percentuais nos extremos do período. A violência sexual foi a mais frequente, seguida de maus tratos e violência física. Contudo, enquanto a violência sexual apresentou a mesma tendência de declínio no primeiro ano da pandemia, com flutuações positivas e negativas, houve um incremento na taxa de maus tratos em 2020 (11,7 vs. 18,16) no primeiro ano da pandemia.

**Tabela 3:** Frequência de casos notificados e taxa por tipo de violência doméstica em meninas menores de 18 anos por 100.000 habitantes na cidade de Maputo, Moçambique, 2019-2022

Tipo de violência	Meninas			
	2019	2020	2021	2022
	n* (taxa)	n* (taxa)	n* (taxa)	n* (taxa)
Violência física	22 (9,90)	10 (4,57)	15 (6,94)	3 (1,40)
Violência psicológica	3 (1,35)	2 (0,91)	3 (1,39)	0
Violência financeira	1 (0,45)	0	2 (0,93)	0
Violência sexual	118 (53,10)	91 (41,57)	99 (45,81)	81 (37,9)
Maus tratos	26 (11,7)	41 (18,16)	18 (8,33)	20 (9,36)
Total	170 (76,5)	144 (65,78)	137 (63,40)	104 (48,66)

*n = Número de casos notificados

A Tabela 4 apresenta a frequência de casos notificados e a taxa de violência doméstica em mulheres adultas, por tipo de violência, por 100.000 habitantes. Observou-se um decréscimo na taxa global de violência ao longo do período, de 594,19 em 2019 para 213,94 por 100.000 mulheres em 2022. A violência física foi o tipo mais frequente, seguida pela psicológica e financeira. As taxas de violência sexual mantiveram-se estáveis em 2019 (14,23) e 2020 (13,52), quando, então, caíram abruptamente em 2021 (2,98) com um aumento em 2021 (4,45). Maus tratos foram incomuns em mulheres em 2019 e 2020, mas em 2021 observou-se uma taxa 14,92 por 100 mil mulheres. 

**Tabela 4:** Frequência de casos notificados e taxa por tipo de violência doméstica em mulheres adultas por 100.000 habitantes na cidade de Maputo, Moçambique, 2019-2022

Tipo de violência	Adultas			
	2019	2020	2021	2022
	n* (taxa)	n* (taxa)	n* (taxa)	n* (taxa)
Violência física	1398 (423,38)	1134 (340,64)	744 (221,98)	380 (112,77)
Violência psicológica	381 (115,39)	249 (74,8)	298 (88,91)	263 (78,05)
Violência financeira	133 (40,28)	90 (27,03)	74 (22,08)	52 (15,41)
Violência sexual	47 (14,23)	45 (13,52)	10 (2,98)	15 (4,45)
Maus tratos	3 (0,91)	0	50 (14,92)	11 (3,26)
Total	1962 (594,19)	1518 (455,99)	1176 (350,87)	721 (213,96)

*n = Número de casos notificados

Em 2019 a taxa global de notificações de violência em idosas foi de 369,71 por 100 mil idosas, observando-se um declínio substancial em 2021 (45,81) e um aumento em 2022 as custas de maus tratos (Tabela 5). A violência física e a psicológica foram as mais frequentes em 2019, mas seguiram ao longo da pandemia em declínio, com nenhum caso registrado em 2022. Ao contrário, a taxa de violência financeira e, mais acentuadamente, os maus tratos aumentaram aproximadamente em 40% em 2020. Em 2021, a taxa de violência financeira declinou para 7,05, mantendo-se em 10 casos com leve aumento em 2022. Por outro lado, não houve registro de maus tratos em 2021, mas em 2022 a taxa chegou a 153,37 casos por 100.000 idosas.

**Tabela 5:** Frequência de casos notificados e taxa por tipo de violência doméstica em idosas por 100.000 habitantes na cidade de Maputo, Moçambique, 2019-2022

Tipo de violência	Idosas			
	2019	2020	2021	2022
	n* (taxa)	n* (taxa)	n* (taxa)	n* (taxa)
Violência física	30 (117,99)	16 (59,57)	4 (14,09)	0
Violência psicológica	32 (125,86)	10 (37,23)	3 (10,57)	0
Violência financeira	16 (62,93)	24 (89,36)	2 (7,05)	3 (10,0)
Violência sexual	2 (7,87)	0	4 (14,09)	1 (3,33)
Maus tratos	14 (55,06)	50 (186,16)	0	46 (153,37)
Total	94 (369,71)	100 (372,33)	13 (45,81)	50 (166,71)

*n = Número de casos notificados

O modelo de regressão de Poisson com variância robusta mostrou que no período pandêmico houve uma redução de 42% (RTI: 0,58; IC 95%: 0,43-0,79) na taxa de incidência de violência quando comparada ao período pré-pandêmico, indicando um impacto significativo da pandemia de COVID-19 na diminuição das taxas. Adicionalmente, a análise revelou que mulheres adultas (18-59 anos) apresentaram uma taxa de incidência 535% maior (RTI: 6,35; IC95%: 4,78-8,43), enquanto mulheres idosas (>60 anos) tiveram uma taxa 270% maior (RTI: 3,70; IC95%: 2,14-6,40), ambas comparadas à faixa etária de 0-17 anos (crianças e adolescentes).

## Discussão

O presente estudo ecológico analisou os casos de violência doméstica em meninas, mulheres e idosas no período pré-pandêmico (2019) e pandêmico (2020-2022) na Cidade de Maputo, Moçambique. Essa abordagem foi fundamental para entender as mudanças nos níveis de violência antes e durante a pandemia de COVID-19, além de observar variações nos tipos de violência e entre diferentes grupos etários. A análise permitiu identificar tendências importantes, como a redução de casos notificados durante a pandemia e a variação no impacto de diferentes tipos de violência em grupos etários distintos, fornecendo uma visão abrangente dos efeitos da crise de saúde pública sobre a violência doméstica.

Estimativas de violência contra mulher no continente africano têm mostrado frequências elevadas, e o presente estudo ratifica este cenário em Maputo, capital de Moçambique^2^. Mesmo com a diminuição nas notificações ao longo dos anos estudados, a quantidade de casos de violência física, psicológica, sexual e maus tratos é significativa, o que ratifica uma realidade alarmante. 

Em Moçambique, dados do Inquérito Demográfico e de Saúde de 2011^12^ revelaram que 37% das mulheres com idade igual ou superior a 15 anos foram vítimas de violência física e/ou sexual ao longo da vida, sendo quase a totalidade dos casos perpetradas pelos parceiros. A cultura local naturaliza a violência contra mulher e isso certamente contribui para o cenário apresentado neste estudo. De acordo com o mesmo documento, 23% das mulheres justificaram pelo menos uma razão para que o esposo agrida a esposa, e 19% dos homens pelo menos uma razão que justificaria a agressão do marido em relação à esposa^12^.

No contexto da pandemia, as medidas de quarentena adotadas para conter a COVID-19, embora fundamentais para a proteção da saúde pública, tiveram consequências inesperadas, como o aumento significativo da violência doméstica, que afetou de forma desproporcional as mulheres^6^. O isolamento social, necessário para reduzir o contágio, criou um paradoxo: ao mesmo tempo em que as pessoas foram isoladas fisicamente para se protegerem, muitas ficaram mais vulneráveis dentro de suas próprias casas^7^.

Para alguns autores, o confinamento deu mais liberdade aos agressores, aumentando os casos de violência em diversos países, como Austrália, China, em diferentes estados dos Estados Unidos da América e até no Reino Unido^16^. O isolamento doméstico impediu que muitas vítimas tivessem acesso a ajuda, o que intensificou sua vulnerabilidade. 

Entretanto, o presente estudo mostra que entre 2019 e 2022, houve uma redução de aproximadamente 60% no número de casos notificados de violência contra pessoas do sexo feminino na cidade de Maputo. A violência física foi a mais comum, seguida da psicológica e sexual. A análise por faixa etária mostrou que mulheres adultas e idosas enfrentaram principalmente violência física e psicológica, enquanto as meninas foram mais vítimas de violência sexual. No caso das idosas, houve um aumento em maus tratos, especialmente em 2022. Além disso, a análise de regressão de Poisson indicou que mulheres adultas e idosas (RPA: 6,35 e RPA: 3,7) têm maior risco de sofrer violência do que meninas e que a incidência de violência foi menor no período pandêmico comparado ao período pré-pandêmico. 

Embora tenha havido uma queda, o número de casos de violência doméstica foi expressivo no ano anterior à pandemia, demonstrando que a violência contra pessoas do sexo feminino é um problema importante na cidade, especialmente entre mulheres adultas e que o declínio de casos ocorreu muito provavelmente devido à subnotificação. Fatores como o medo de denunciar, aliado ao temor das represálias por parte da família e da sociedade e a dependência financeira associada à baixa escolaridade e rendimentos limitados são motivos para que as mulheres desistam de formalizar suas denúncias^17^. Esses fatores criam um contexto em que as barreiras à denúncia se tornam mais fortes, agravando a subnotificação de casos de violência.

A omissão das notificações de casos de violência é um obstáculo significativo no enfrentamento desse problema. Ao impedir que a violência seja devidamente registrada e visível, dificulta-se a formulação de políticas públicas de prevenção e a oferta de serviços de apoio às vítimas. Esse cenário prejudica a capacidade das autoridades e instituições de agir de forma eficaz para interromper o ciclo de violência e oferecer a proteção necessária^18^.

Um estudo conduzido em Maputo^19^, também no período pré-pandêmico (abril de 2019) e anterior à Declaração de Estado de Emergência (março de 2020) e trans pandêmico (maio a junho de 2020), revelou uma tendência de aumento de número de violências, sendo notificados 83 casos em abril de 2019 e 169 casos em março de 2020. 

No entanto, como apresentado neste estudo, em maio, o número de casos declinou para 131 casos notificados. Segundo este estudo, a aparente redução não deve refletir a realidade, uma vez que, em Moçambique, as denúncias de violência nas PRM e GAFMVV são feitas de forma presencial, ratificando a suspeição de subnotificação.

Assim, a redução nas notificações ao longo do período investigado pode ser menos um reflexo da diminuição real da violência e mais uma consequência de fatores como o medo de denunciar, a dependência financeira ou a menor acessibilidade a serviços de apoio, sugerindo, dessa forma, que, embora os números tenham diminuído, a violência continuava em níveis elevados.

Violência sexual foi frequente em meninas com menos de 18 anos. A referida faixa etária é muito ampla e os dados não nos permitiram desagregá-la em faixas etárias menores, de forma a possibilitar que identificássemos quais intervalos de idade são predominantes nesses tipos de violência. Hipotiza-se que a violência sexual pode ter ocorrido de forma mais frequente em adolescentes, e os maus tratos podem ocorrer mais entre crianças ainda dependentes dos cuidados de pais ou responsáveis. 

Maus tratos foram mais frequentes em meninas e idosas. A restrição de mobilidade, o isolamento social, o aumento das tensões familiares podem deixar as crianças mais irritadas, levando a comportamentos agressivos e de desobediência, elevando, desse modo, o risco de violência doméstica^20^. No caso de idosas, a maior dependência de terceiros para realizar tarefas básicas, o limitado apoio social devido ao isolamento, somado à insuficiência de mecanismos e formas de denúncia^21^ são fatores que podem ter levado à subnotificação e flutuações dos casos de violência nessas faixas etárias na cidade de Maputo.

Em idosas, houve também um incremento de violência financeira no primeiro ano da pandemia, com subsequente declínio. As limitações de mobilidade impostas pela pandemia da COVID-19 impactaram a economia mundial^22^ ^)^ e consequentemente a economia doméstica. Assim, idosos podem ter sofrido abusos considerando seus bens, pensões e aposentadorias para a manutenção da família. 

A redução da taxa de incidência de violência na cidade de Maputo, observada na análise de regressão de Poisson, indica uma possível subnotificação durante o período pandêmico e não necessariamente um efeito protetor real na incidência de violência doméstica durante o período pandêmico (2020-2022) em comparação com o período pré-pandêmico (2019). 

Embora à primeira vista essa redução possa ser vista como positiva, a interpretação desse efeito é complexa e pode refletir vários fatores contextuais e estruturais^23^. A verdadeira dimensão da violência pode estar mascarada por vários fatores além da subnotificação, como mudanças nos tipos de violência prevalentes e restrições ao acesso a serviços de apoio e proteção, sugerindo a necessidade de melhorar os sistemas de notificação e garantir que as vítimas tenham acesso aos recursos necessários, independentemente das condições externas, como a pandemia.

Os resultados sobre a violência doméstica contra mulheres em seus ciclos de vida na cidade de Maputo entre 2019 e 2022 podem ser aplicados na prática para direcionar diversas intervenções e mudanças nas áreas de saúde, assistência social e política públicas.

Algumas ações práticas podem ser implementadas para enfrentar o problema da violência doméstica^5^, como:

Fortalecimento dos serviços de saúde e notificação para melhorar a identificação, atendimento e notificação, incluindo a capacitação de profissionais de saúde para identificar sinais de violência, mesmo que as vítimas não relatem diretamente; o estabelecimento de protocolos de triagem sistemática em todas as consultas de saúde, ginecológicos, pediátricos e geriátricos; e aperfeiçoamento do registro de dados e notificação para assegurar que todos os casos identificados sejam registrados e reportados às autoridades;

- Desenvolvimento de redes de apoio integradas que ofereçam assistência completa às mulheres em situação de violência, criando centros de acolhimento onde as pessoas em situação de violência possam buscar ajuda imediata e obter apoio legal, psicológico e médico, que sejam acessíveis e operem 24 horas por dia;
- Programas de conscientização e educação comunitária, implementando ações como: programas educativos para educar as comunidades sobre os diferentes tipos de violência (física, psicológica, financeira, maus tratos e sexual) e as maneiras de denunciar, por meio de campanhas de mídia, palestras em escolas e centros comunitários e programas de rádio; incentivar o empoderamento das mulheres, especialmente as mais vulneráveis, por meio de programas de educação, qualificação profissional e apoio econômico, pois mulheres com maior independência têm maior capacidade de denunciar os agressores e de sair de relações abusivas;
- Implementação de serviços de apoio remoto para garantir que as mulheres em situação de violência tenham apoio mesmo em situações de restrição de mobilidade, alocando telefones de emergência gratuitos ou aplicativos que permitam a denúncia anônima; e sites ou aplicativos onde as vítimas podem acessar informações sobre como procurar ajuda, além de oferecer suporte emocional através de bate-papos com profissionais de saúde mental;
- Intervenções focadas em faixas etárias específicas para responder às necessidades de cada grupo, focando em programas de prevenção de violência sexual e maus tratos, equipando as escolas com programas de educação sobre direitos, autocuidado e como denunciar; oferecer programas que abordem a violência física e psicológica, com foco em estratégias de resiliência, suporte emocional e econômico; e desenvolver intervenções específicas para o combate ao abuso e maus tratos de idosas, incluindo campanhas de conscientização sobre abuso financeiro e negligência e a criação de serviços de apoio especializado;
- Monitoramento contínuo e políticas baseadas em evidências: os governos locais e nacionais devem usar os dados das notificações e pesquisas sobre violência para monitorar tendências e desenvolver políticas públicas baseadas em evidências que atendam às realidades da população feminina em diferentes contextos;
- Capacitação contínua para profissionais da saúde e assistência social que atuam diretamente com vítimas, com treinamentos periódicos sobre as melhores práticas para lidar com casos de violência, técnicas de acolhimento e procedimentos legais fundamentais para melhorar a resposta dos serviços de saúde e sociais; e programas de formação que enfatizam a necessidade de tratar casos de violência de forma sensível e adaptada a diferentes contextos culturais, econômicos e sociais;

Com base nos resultados apresentados, sugere-se que futuras pesquisas abordem temas como:

- Barreiras ao registro de denúncias: investigar como as medidas de isolamento durante a pandemia afetaram a capacidade das mulheres de denunciar a violência, considerando fatores como falta de acesso a dispositivos eletrônicos e a baixa familiaridade com tecnologias em populações vulneráveis; e avaliar como ampliar o acesso e a efetividade de canais de denúncia alternativos, como plataformas online ou serviços telefônicos;
- Violência contra idosas e mudança nos padrões de violência: investigar as causas por trás do aumento dos casos de maus tratos em idosas e a diminuição dos casos de violência física e psicológica; e compreender os fatores que levam à vulnerabilidade financeira e maus tratos;
- Violência em meninas: analisar mais detalhadamente o aumento de maus tratos e a variação de casos de violência sexual em meninas menores de 18 anos, identificando medidas preventivas e de proteção mais eficazes para essa faixa etária;
- Determinantes sociais e culturais da violência: investigar sobre os fatores sociais, culturais e econômicos que contribuem para a violência contra mulheres na cidade de Maputo; e estudar como a pobreza, o desemprego, normas de gênero e a estrutura familiar influenciam as dinâmicas da violência, além de explorar como essas variáveis afetam as mulheres nos diferentes ciclos de vida.

As limitações do estudo incluem aspectos relacionados à qualidade dos registros em Maputo, os quais apresentaram variações ao longo dos quatro anos estudados, dificultando uma análise mais precisa. Adicionalmente, os dados fornecidos foram dados agregados dos sete distritos municipais da cidade de Maputo, o que impediu a análise territorial das ocorrências. A ausência de informações sobre o perfil demográfico das vítimas e dos agressores restringiu a compreensão das dinâmicas dos casos. Por fim, as variações na forma de agregação dos dados por faixa etária dificultaram a comparação direta entre os diferentes grupos etários.

## Conclusão

A análise da violência contra pessoas do sexo feminino na cidade de Maputo, Moçambique, entre 2019 e 2022, revela padrões importantes em termos de variações etárias, temporais e contextuais, como a pandemia. As mulheres adultas foram o grupo mais impactado pela violência ao longo dos anos, refletindo exposição elevada a diferentes formas de violência, como física, psicológica e financeira. As idosas também apresentaram uma taxa de violência considerável, sendo mais propensas a sofrer abusos do que as crianças, com destaque para o aumento dos maus tratos nos anos de 2020 e 2022. Durante o período pandêmico, observou-se uma redução de 42% nas notificações de casos de violência em relação ao período pré-pandêmico. Essa diminuição pode estar associada ao confinamento social, às mudanças nas rotinas diárias e à redução na capacidade de denúncia, além do impacto socioeconômico causado pela pandemia, que alterou as dinâmicas familiares e sociais, dificultando a identificação e o relato dos casos de violência. Assim, essa redução deve ser analisada com cautela, pois pode não refletir uma diminuição real da violência, mas sim uma menor notificação dos casos devido às restrições e à dificuldade de acesso aos serviços de proteção.

Mesmo tendo havido redução dos casos durante a pandemia, é fundamental monitorar o cenário pós-pandêmico para entender se a tendência de redução se mantém ou se novos fatores surgem que possam aumentar a violência, capacitar profissionais de saúde, na educação e assistência social para identificar e intervir nos casos de violência, além de sensibilizar a população para o problema, continuar com a coleta de dados e monitoramento dos casos de violência, como estratégia importante para identificar tendências emergentes no combate à violência de forma eficaz.

Em suma, embora os dados apontem para uma tendência de redução da violência contra mulheres na Cidade de Maputo, ainda há desafios a serem enfrentados, especialmente em relação aos maus tratos e à violência sexual contra meninas. O fortalecimento das redes de apoio e a promoção de políticas de prevenção, juntamente com a consolidação de sistemas de vigilância e proteção social, são fundamentais para abordar esse grave problema social, e garantir a segurança e o bem-estar da população feminina, promovendo um ambiente mais seguro e acolhedor.

## Declaração de Disponibilidade de Dados:

Os conjuntos de dados relacionados a este artigo estarão disponíveis mediante solicitação ao autor correspondente.

# Translation: Impacto de la pandemia de COVID-19 en la violencia doméstica, 2019-2022^3^

**DOI:** 10.1590/1518-8345.7868.4743

**Authors:** Teresa Mutemba Vilanculo, Leonora Rezende Pacheco, Karlla Antonieta Amorim Caetano, Rafael Alves Guimarães, Sheila Araújo Teles

**Affiliations:** 1 Universidade Federal de Goiás, Faculdade de Enfermagem, Goiânia, GO, Brasil.; 2 Becaria de la Coordenação de Aperfeiçoamento de Pessoal de Nível Superior (CAPES), Brasil.

**Keywords:** Violencia Doméstica, Mujer, Niño, Anciano, Pandemias, COVID-19

## Abstract

**Objetivo:**

analizar la incidencia y los tipos de violencia doméstica contra niñas, mujeres adultas y mayores, antes y durante la pandemia de COVID-19

**Método:**

estudio ecológico. Se calcularon tasas de incidencia global y por tipo de violencia y se realizó regresión de Poisson para evaluar el impacto de la pandemia de COVID-19 sobre la tasa de violencia contra niñas, mujeres adultas y mayores.

**Resultados:**

se verificó una frecuencia elevada de violencia doméstica en el período pre y trans pandemia, con una tasa media general de 235,32 casos/100.000 habitantes. Sin embargo, se observó una reducción de 383,5/100.000 (período prepandemia) a 150,7/100.000 (período trans pandemia). Los datos por ciclo de vida mostraron que, a pesar del descenso, la violencia sexual y la violencia física fueron las más frecuentes en niñas y en mujeres, respectivamente. En mujeres mayores, los malos tratos y la violencia financiera aumentaron en el primer año de la pandemia. Las mujeres adultas y las mayores presentaron mayor riesgo de violencia, 6,35 y 3,7 veces en comparación con las niñas.

**Conclusión:**

la violencia doméstica es un grave problema de salud pública. La reducción de casos durante la pandemia sugiere subnotificación debido a las restricciones impuestas por la pandemia.

## Destacados

**(1)** La violencia contra la mujer es un problema de salud pública en Maputo, Mozambique. **(2)** Las mujeres adultas en Maputo son las más vulnerables a la violencia doméstica. **(3)** Hubo reducción en la notificación de casos de violencia contra las mujeres durante la pandemia.

## Introducción

La violencia interpersonal es un fenómeno complejo y multicausal de salud pública, responsable de alta morbilidad, incluidas discapacidades, hospitalizaciones y mortalidad^1^ ^-^ ^2^. El impacto de la violencia va más allá de las víctimas directas, afectando también al sistema de salud y a la economía, que enfrentan una sobrecarga creciente debido a la demanda de asistencia en salud y social^1^.

La violencia interpersonal incluye la violencia familiar o por pareja íntima, también denominada violencia doméstica, que afecta a niños, mujeres y personas mayores^1^. Aunque la violencia ocurre en ambos sexos, las mujeres están desproporcionadamente afectadas por la violencia interpersonal. Globalmente, se estima que 26% de las mujeres de 15 años o más ya sufrió violencia física y/o sexual por pareja íntima en algún momento de la vida, mientras que la prevalencia de violencia, incluidos agresores no íntimos, alcanza 30%^2^.

La violencia contra niños incluye todas las formas de violencia perpetradas por padres, familiares, cuidadores, pareja romántica o cualquier otra persona de su círculo social o no. Una revisión sistemática estimó que 54%, es decir, más de mil millones de niños de 2 a 17 años, sufrieron alguna forma de violencia en los últimos 12 meses^3^. A su vez, la violencia contra personas mayores, también denominada abuso de personas mayores, afecta a 15,7% de los mayores no institucionalizados a nivel global, siendo el abuso psicológico y financiero las principales formas de violencia^4^.

Considerando la magnitud de la violencia como problema de salud pública, la Organización de las Naciones Unidas (ONU) incluyó la eliminación de la violencia contra mujeres y niños en los Objetivos de Desarrollo Sostenible (ODS) de la Agenda 2030. Además, la Organización Mundial de la Salud (OMS) refuerza el papel del sistema de salud en políticas destinadas al enfrentamiento de la violencia, por medio del *Plan de Acción Global para fortalecer el papel del sistema de salud dentro de una respuesta multisectorial dirigida a la violencia interpersonal, en especial la violencia contra mujeres y niños*. El documento destaca, asimismo, la necesidad de datos sólidos para fundamentar políticas públicas eficaces^5^.

En 2020, la pandemia de COVID-19 impuso restricciones de movilidad y reglas de distanciamiento social que resultaron en una mayor convivencia doméstica. Sin embargo, el hogar puede no ser el lugar más seguro para personas que sufren violencia doméstica y el escenario pandémico contribuyó al aumento de casos de ese tipo de violencia^6^ ^-^ ^7^. En Estados Unidos de América^8^, investigadores mostraron que, justo después de la implementación de esas medidas, hubo un aumento de la violencia doméstica, que varió de 10% en Nueva York a 27% en Jefferson, Alabama. En países europeos, hubo un aumento de 60% de llamadas de emergencia por violencia doméstica en 2020 en comparación con 2019^9^.

En el continente africano, se estima que el 33% de las mujeres ya sufrió alguna forma de violencia en la vida^2^. En Mozambique, la violencia doméstica es una cuestión persistente, agravada por la cultura patriarcal y por la escasez de recursos para la denuncia y el apoyo a las víctimas^10^. Aunque el gobierno mozambiqueño y entidades no gubernamentales han hecho un gran esfuerzo para fortalecer la legislación contra la violencia doméstica y mejorar la calidad de vida de las personas sobrevivientes^11^, aún hay poca información sobre violencia doméstica en ese país y, en particular, sobre el impacto de la pandemia en los casos de violencia. Esa información es fundamental para contribuir a los esfuerzos de construcción de políticas públicas de enfrentamiento de este agravio a la salud, principalmente en períodos de crisis sanitarias, como la de la pandemia de COVID-19.

El objetivo del presente estudio fue analizar la incidencia y los tipos de violencia doméstica contra niñas, mujeres adultas y mayores en la ciudad de Maputo, antes y durante la pandemia de COVID-19 (2019-2022). 

## Método

### Diseño del estudio

Se trata de un estudio ecológico retrospectivo, con un enfoque cuantitativo y componentes descriptivos y analíticos. 

### Lugar

El estudio fue conducido en la ciudad de Maputo, capital de Mozambique. Según el censo demográfico de 2017, Mozambique posee una población de 26.899.105 habitantes y un Índice de Desarrollo Humano (IDH) de 0,456, posicionándose como el noveno peor entre los 189 países evaluados por el Programa de las Naciones Unidas para el Desarrollo^12^ ^-^ ^13^. En Maputo, la población estimada es de 1.118.387, siendo 576.488 mujeres^12^.

### Período

El período de selección (recorte) de la demanda analizada fue del 01 de enero de 2019 al 31 de diciembre de 2022. La recolección de datos se realizó del 02 de enero al 30 de junio de 2023.

### Población

La población del estudio estuvo compuesta por los casos notificados de violencia doméstica contra niñas, mujeres adultas y mayores residentes en la ciudad de Maputo, Mozambique.

### Variables del estudio

Se analizaron los siguientes indicadores:

(i) Tasa de violencia doméstica: el indicador se calcula por la razón entre el número absoluto de casos de violencia en niñas, adultas y mayores y la población residente correspondiente de Maputo, multiplicado por 100.000.

(ii) Tasa de violencia doméstica por tipología: el indicador se calcula por la razón entre el número absoluto de casos de violencia por tipología en niñas, adultas y mayores y la población residente correspondiente de Maputo, multiplicado por 100.000.

En este trabajo, se analizaron los actos criminales de violencia contra niñas, adultas y mayores, descritos en la Ley n.º 29/2009 de 29 de septiembre de 2009, denominada Ley de Violencia Doméstica perpetrada contra la Mujer, conforme se sigue: violencia física simple, violencia física grave, violencia psicológica, violencia moral, cópula no consentida, cópula con transmisión de enfermedad, violación, malos tratos.

La definición de esos tipos de violencia se describe en la Ley n.º 29/2009^14^. A los fines de este estudio, violencia física simple y grave se agruparon y denominaron “violencia física”; mientras que violencia psicológica y moral se agruparon y denominaron “violencia psicológica”; por su parte, cópula no consentida, cópula con transmisión de enfermedad y violación se agruparon y denominaron “violencia sexual”; mientras que violencia patrimonial se denominó “violencia financiera”. 

### Recolección de datos

Los datos fueron extraídos de registros del Departamento de Atención a la Familia y Menores Víctimas de Violencia (DAFMVV), que compilan los datos de notificaciones de violencias en los siete distritos municipales de la ciudad de Maputo. En Mozambique, las notificaciones son realizadas presencialmente por la víctima en las comisarías de la Policía de la República de Mozambique (PRM) o en el Gabinete de Atención a la Familia y Menores Víctimas de Violencia (GAFMVV) de Maputo. En casos de menores de edad o personas con limitaciones físicas o cognitivas, el responsable legal realiza la notificación.

Además de los registros de las comisarías de la policía, se utilizaron las proyecciones poblacionales para 2019, 2020, 2021 y 2022, a partir del último censo realizado en la República de Mozambique en 2017, conducido por el Instituto Nacional de Estadística (INE).

El informe del DAFMVV presenta datos agrupados por edad, considerando tres periodos de vida: niñas (0-17 años), adultas (18-59 años) y mayores (≥ 60 años).

### Tratamiento y análisis de datos

Los datos fueron analizados utilizando el paquete estadístico STATA, versión 17,0. Se presentaron las frecuencias absolutas y las tasas de violencia doméstica global y estratificadas por tipo de violencia y franja etaria para los años 2019, 2020, 2021 y 2022.

La unidad de análisis en este estudio incluye las notificaciones de violencia contra niñas, adultas y mayores que fueron agregadas de acuerdo con el año de notificación, la tipología de la violencia (física, psicológica, patrimonial, sexual y malos tratos), el número total de casos registrados y la franja etaria de las víctimas.

Se utilizó un modelo de regresión de Poisson con varianza robusta para evaluar el impacto de la pandemia de COVID-19 sobre la tasa de violencia contra la mujer. La variable dependiente del modelo fue la tasa de violencia (por 100.000 mujeres), y las variables independientes incluyeron la franja etaria (0-17 años, 18-59 años y >60 años) y una variable *dummy*, en la que “0” representó el período prepandémico (del 01/01/2019 al 31/12/2019) y “1”, el período pandémico (del 01/01/2020 al 31/12/2022). Los resultados del modelo se expresaron como Razón de Tasa de Incidencia (RTI) y sus respectivos intervalos de confianza del 95% (IC95%). La significancia estadística se evaluó mediante la prueba de Wald, adoptándose p < 0,05 como umbral de significancia^15^.

### Aspectos éticos

Se utilizaron datos secundarios de dominio público, obtenidos del Departamento de Atención a la Familia y Menores Víctimas de Violencia, en la Ciudad de Maputo, Mozambique, y del Instituto Nacional de Estadística. Por tratarse de datos agregados que no contienen informaciones personales identificables de la población en estudio, no hubo necesidad de obtener un Consentimiento Informado (CI). Así, se cumplieron las determinaciones de la Resolución n.º 510, de 7 de abril de 2016, y la Ley n.º 6/2023, Ley de Investigación en Salud Humana de la República de Mozambique.

### Resultados

La Tabla 1 presenta las tasas de violencia doméstica por 100.000 habitantes en la ciudad de Maputo, Mozambique, entre 2019 y 2022. Las tasas totales disminuyeron a lo largo de los cuatro años, de 383,50 por 100.000 habitantes en 2019 a 150,7 en 2022, reflejando una caída pronunciada en las tasas de violencia reportadas. La violencia física fue el tipo de violencia más frecuente, seguida de la violencia psicológica. 

**Tabla 1:** Tasas de violencia doméstica en niñas, mujeres adultas y mayores, por tipología, por 100.000 habitantes en la ciudad de Maputo, Mozambique, 2019-2022

Tipo de violencia	Año			
	2019	2020	2021	2022
	n*/Tasa	n*/Tasa	n*/Tasa	n*/Tasa
Violencia física	1450/250,93	1160/200,46	763/131,64	383/65,96
Violencia psicológica	416/71,99	261/45,1	301/52,45	263/45,29
Violencia financiera	140/25,96	114/19,7	76/13,46	55/9,47
Violencia sexual	167/28,9	136/23,5	113/19,5	97/16,7
Malos tratos	43/15,73	91/13,26	68/11,73	77/7,44
Total	2216/383,50	1762/304,49	1321/227,91	875/150,69

*n = Número de casos notificados

Las mujeres fueron víctimas principalmente de violencia física y psicológica, con tasas medias de 271,69 y 89,28, respectivamente. En niñas, la mayor tasa media fue de violencia sexual (44,60), seguida de malos tratos (12,3). Por otro lado, en mujeres mayores, la tasa media más alta fue la de malos tratos (98,65), seguida de violencia física (47,92), psicológica (43,42) y financiera (42,33) (Tabla 2).

**Tabla 2:** Tasa media global y por tipo de violencia doméstica en niñas, mujeres adultas y mayores en la Ciudad de Maputo, Mozambique, 2019-2022

Tipo de violencia	Niñas (IC95%)*	Adultas (IC95%)*	Mayores (IC 95%)*	Todas las franjas etarias (IC95%)*
Física	5,70 (-0,28-11,43)	271,69 (58,35-491,04)	47,92 (-36,72-132,55)	109,44 (17,28-201,59)
Psicológica	0,91 (-0,11-1,94)	89,28 (59,98-118,59)	43,42 (-47,53-134,36)	44,54 (13,39-75,69)
Financiera	0,34 (-0,36-1,05)	26,21 (9,47-42,94)	42,33 (-22,14-106,81)	22,96 (4,94-40,98)
Sexual	44,60 (34,21-54,98)	8,80 (-0,60-18,19)	6,32 (-3,39-16,03)	19,91 (7,77-32,04)
Malos tratos	12,03 (4,58-19,48)	4,77 (-6,2-15,76)	98,65 (-38,49-235,79)	38,48 (-1,85-78,81)
Total	63,59 (45,34-81,83)	403,75 (147,48-660,02)	238,64 (-16,97-494,25)	235,32 (16,13-354,52)

*IC95% = Intervalo de Confianza del 95%

La Tabla 3 presenta la frecuencia de casos notificados y la tasa de violencia doméstica en niñas, por tipo de violencia, por 100.000 habitantes, entre 2019 y 2022. En 2019 se notificaron 170 casos de violencia contra niñas menores de 18 años, observándose una reducción en los extremos del período de 20 puntos porcentuales. La violencia sexual fue la más frecuente, seguida de malos tratos y violencia física. No obstante, mientras la violencia sexual mostró una tendencia de descenso en el primer año de la pandemia, con fluctuaciones positivas y negativas, hubo un incremento en la tasa de malos tratos en 2020 (11,7 vs. 18,16) en el primer año de la pandemia.

**Tabla 3:** Frecuencia de casos notificados y tasa por tipo de violencia doméstica en niñas menores de 18 años por 100.000 habitantes en la ciudad de Maputo, Mozambique, 2019-2022

Tipo de violencia	Niñas			
	2019	2020	2021	2022
	n* (tasa)	n* (tasa)	n* (tasa)	n* (tasa)
Violencia física	22 (9,90)	10 (4,57)	15 (6,94)	3 (1,40)
Violencia psicológica	3 (1,35)	2 (0,91)	3 (1,39)	0
Violencia financiera	1 (0,45)	0	2 (0,93)	0
Violencia sexual	118 (53,10)	91 (41,57)	99 (45,81)	81 (37,9)
Malos tratos	26 (11,7)	41 (18,16)	18 (8,33)	20 (9,36)
Total	170 (76,5)	144 (65,78)	137 (63,40)	104 (48,66)

*n = Número de casos notificados

La Tabla 4 presenta la frecuencia de casos notificados y la tasa de violencia doméstica en mujeres adultas, por tipo de violencia, por 100.000 habitantes. Se observó un descenso en la tasa global de violencia a lo largo del período, de 594,19 en 2019 a 213,94 por 100.000 mujeres en 2022. La violencia física fue el tipo más frecuente, seguida por la psicológica y la financiera. Las tasas de violencia sexual se mantuvieron estables en 2019 (14,23) y 2020 (13,52), cuando, entonces, cayeron abruptamente en 2021 (2,98) con un aumento en 2021 (4,45). Los malos tratos fueron poco comunes en mujeres en 2019 y 2020, pero en 2021 se observó una tasa de 14,92 por 100 mil mujeres. 

**Tabla 4:** Frecuencia de casos notificados y tasa por tipo de violencia doméstica en mujeres adultas por 100.000 habitantes en la ciudad de Maputo, Mozambique, 2019-2022

Tipo de violencia	Adultas			
	2019	2020	2021	2022
	n* (tasa)	n* (tasa)	n* (tasa)	n* (tasa)
Violencia física	1398 (423,38)	1134 (340,64)	744 (221,98)	380 (112,77)
Violencia psicológica	381 (115,39)	249 (74,8)	298 (88,91)	263 (78,05)
Violencia financiera	133 (40,28)	90 (27,03)	74 (22,08)	52 (15,41)
Violencia sexual	47 (14,23)	45 (13,52)	10 (2,98)	15 (4,45)
Malos tratos	3 (0,91)	0	50 (14,92)	11 (3,26)
Total	1962 (594,19)	1518 (455,99)	1176 (350,87)	721 (213,96)

*n = Número de casos notificados

En 2019, la tasa global de notificaciones de violencia en mujeres mayores fue de 369,71 por 100.000 mujeres mayores, observándose un descenso sustancial en 2021 (45,81) y un aumento en 2022 a expensas de malos tratos (Tabla 5). La violencia física y psicológica fueron las más frecuentes en 2019, pero continuaron en descenso a lo largo de la pandemia, sin ningún caso registrado en 2022. Por el contrario, la tasa de violencia financiera y, más acentuadamente, los malos tratos aumentaron aproximadamente un 40% en 2020. En 2021, la tasa de violencia financiera disminuyó a 7,05, manteniéndose en 10 casos con un leve aumento en 2022. Por otro lado, no hubo registro de malos tratos en 2021, pero en 2022 la tasa alcanzó 153,37 casos por 100.000 mujeres mayores.

**Tabla 5:** Frecuencia de casos notificados y tasa por tipo de violencia doméstica en mujeres mayores por 100.000 habitantes en la ciudad de Maputo, Mozambique, 2019-2022

Tipo de violencia	Mayores			
	2019	2020	2021	2022
	n* (tasa)	n* (tasa)	n* (tasa)	n* (tasa)
Violencia física	30 (117,99)	16 (59,57)	4 (14,09)	0
Violencia psicológica	32 (125,86)	10 (37,23)	3 (10,57)	0
Violencia financiera	16 (62,93)	24 (89,36)	2 (7,05)	3 (10,0)
Violencia sexual	2 (7,87)	0	4 (14,09)	1 (3,33)
Malos tratos	14 (55,06)	50 (186,16)	0	46 (153,37)
Total	94 (369,71)	100 (372,33)	13 (45,81)	50 (166,71)

*n = Número de casos notificados

El modelo de regresión de Poisson con varianza robusta mostró que en el período pandémico hubo una reducción del 42% (RTI: 0,58; IC95%: 0,43-0,79) en la tasa de incidencia de violencia en comparación con el período prepandémico, indicando un impacto significativo de la pandemia de COVID-19 en la disminución de las tasas. Adicionalmente, el análisis reveló que las mujeres adultas (18-59 años) presentaron una tasa de incidencia 535% mayor (RTI: 6,35; IC95%: 4,78-8,43), mientras que las mujeres mayores (>60 años) tuvieron una tasa 270% mayor (RTI: 3,70; IC95%: 2,14-6,40), ambas comparadas con la franja etaria de 0-17 años (niñas y adolescentes).

## Discusión

El presente estudio ecológico analizó los casos de violencia doméstica en niñas, mujeres y mayores en el período prepandémico (2019) y pandémico (2020-2022) en la Ciudad de Maputo, Mozambique. Este abordaje fue fundamental para comprender los cambios en los niveles de violencia antes y durante la pandemia de COVID-19, además de observar variaciones en los tipos de violencia y entre diferentes grupos etarios. El análisis permitió identificar tendencias importantes, como la reducción de casos notificados durante la pandemia y la variación en el impacto de diferentes tipos de violencia en grupos etarios distintos, proporcionando una visión amplia de los efectos de la crisis de salud pública sobre la violencia doméstica.

Las estimaciones de violencia contra la mujer en el continente africano han mostrado frecuencias elevadas, y el presente estudio ratifica este escenario en Maputo, capital de Mozambique^2^. Aun con la disminución en las notificaciones a lo largo de los años estudiados, la cantidad de casos de violencia física, psicológica, sexual y malos tratos es significativa, lo que ratifica una realidad alarmante.

En Mozambique, datos de la Encuesta Demográfica y de Salud de 2011^12^ revelaron que 37% de las mujeres con edad igual o superior a 15 años fueron víctimas de violencia física y/o sexual a lo largo de la vida, siendo casi la totalidad de los casos perpetrados por las parejas. La cultura local naturaliza la violencia contra la mujer y esto ciertamente contribuye al escenario presentado en este estudio. De acuerdo con el mismo documento, 23% de las mujeres justificaron al menos una razón para que el esposo agreda a la esposa, y 19% de los hombres al menos una razón que justificaría la agresión del marido hacia la esposa^12^.

En el contexto de la pandemia, las medidas de cuarentena adoptadas para contener la COVID-19, aunque fundamentales para la protección de la salud pública, tuvieron consecuencias inesperadas, como el aumento significativo de la violencia doméstica, que afectó de forma desproporcionada a las mujeres^6^. El aislamiento social, necesario para reducir el contagio, creó una paradoja: al mismo tiempo que las personas fueron aisladas físicamente para protegerse, muchas quedaron más vulnerables dentro de sus propias casas^7^.

Para algunos autores, el confinamiento dio más libertad a los agresores, aumentando los casos de violencia en diversos países, como Australia, China, en diferentes estados de Estados Unidos de América e incluso en el Reino Unido^16^. El aislamiento doméstico impidió que muchas víctimas tuvieran acceso a ayuda, lo que intensificó su vulnerabilidad. 

Sin embargo, el presente estudio muestra que entre 2019 y 2022 hubo una reducción de aproximadamente 60% en el número de casos notificados de violencia contra personas del sexo femenino en la ciudad de Maputo. La violencia física fue la más común, seguida de la psicológica y la sexual. El análisis por franja etaria mostró que las mujeres adultas y mayores enfrentaron principalmente violencia física y psicológica, mientras que las niñas fueron más víctimas de violencia sexual. 

En el caso de las mujeres mayores, hubo un aumento de malos tratos, especialmente en 2022. Además, el análisis de regresión de Poisson indicó que las mujeres adultas y mayores (RPA: 6,35 y RPA: 3,7) tienen mayor riesgo de sufrir violencia que las niñas y que la incidencia de violencia fue menor en el período pandémico en comparación con el período prepandémico.

Aunque hubo una caída, el número de casos de violencia doméstica fue expresivo en el año anterior a la pandemia, lo que demuestra que la violencia contra personas del sexo femenino es un problema importante en la ciudad, especialmente entre mujeres adultas, y que el descenso de casos ocurrió muy probablemente debido a la subnotificación. Factores como el miedo a denunciar, aliado al temor a represalias por parte de la familia y de la sociedad y a la dependencia financiera asociada a la baja escolaridad y a ingresos limitados, son motivos para que las mujeres desistan de formalizar sus denuncias^17^. Estos factores crean un contexto en el que las barreras a la denuncia se vuelven más fuertes, agravando la subnotificación de casos de violencia.

La omisión de las notificaciones de casos de violencia es un obstáculo significativo en el enfrentamiento de este problema. Al impedir que la violencia sea debidamente registrada y visible, se dificulta la formulación de políticas públicas de prevención y la oferta de servicios de apoyo a las víctimas. Este escenario perjudica la capacidad de las autoridades e instituciones de actuar de forma eficaz para interrumpir el ciclo de violencia y ofrecer la protección necesaria^18^.

Un estudio conducido en Maputo^19^, también en el período prepandémico (abril de 2019) y anterior a la Declaración de Estado de Emergencia (marzo de 2020) y trans pandémico (mayo a junio de 2020), reveló una tendencia de aumento del número de violencias, notificándose 83 casos en abril de 2019 y 169 casos en marzo de 2020. 

Sin embargo, como se presenta en este estudio, en mayo el número de casos disminuyó a 131 casos notificados. Según este estudio, la aparente reducción no debe reflejar la realidad, puesto que, en Mozambique, las denuncias de violencia en las PRM y GAFMVV se realizan de forma presencial, ratificando la sospecha de subnotificación.

Así, la reducción en las notificaciones a lo largo del período investigado puede ser menos un reflejo de la disminución real de la violencia y más una consecuencia de factores como el miedo a denunciar, la dependencia financiera o la menor accesibilidad a servicios de apoyo, sugiriendo, de esa forma, que, aunque los números hayan disminuido, la violencia continuaba en niveles elevados.

La violencia sexual fue frecuente en niñas menores de 18 años. Dicha franja etaria es muy amplia y los datos no nos permitieron desagregarla en franjas de edad menores, de modo que pudiéramos identificar qué intervalos de edad son predominantes en esos tipos de violencia. Así, se hipotetiza que la violencia sexual puede haber ocurrido con mayor frecuencia en adolescentes, y que los malos tratos pueden ocurrir más entre niñas aún dependientes de los cuidados de padres o responsables.

Los malos tratos fueron más frecuentes en niñas y mujeres mayores. La restricción de movilidad, el aislamiento social y el aumento de las tensiones familiares pueden dejar a los niños más irritables, llevando a comportamientos agresivos y de desobediencia, elevando, de este modo, el riesgo de violencia doméstica^20^. En el caso de mujeres mayores, la mayor dependencia de terceros para realizar tareas básicas, el limitado apoyo social debido al aislamiento, sumado a la insuficiencia de mecanismos y vías de denuncia^21^, son factores que pueden haber conducido a la subnotificación y a fluctuaciones de los casos de violencia en esas franjas etarias en la ciudad de Maputo.

En mujeres mayores, hubo también un incremento de violencia financiera en el primer año de la pandemia, con subsiguiente descenso. Las limitaciones de movilidad impuestas por la pandemia de COVID-19 impactaron la economía mundial^22^ y, en consecuencia, la economía doméstica. Así, las personas mayores pueden haber sufrido abusos considerando sus bienes, pensiones y jubilaciones para el sostenimiento de la familia. 

La reducción de la tasa de incidencia de violencia en la ciudad de Maputo, observada en el análisis de regresión de Poisson, indica una posible subnotificación durante el período pandémico y no necesariamente un efecto protector real en la incidencia de violencia doméstica durante el período pandémico (2020-2022) en comparación con el período prepandémico (2019). 

Aunque a primera vista esa reducción pueda verse como positiva, la interpretación de este efecto es compleja y puede reflejar varios factores contextuales y estructurales^23^. La verdadera dimensión de la violencia puede estar enmascarada por diversos factores además de la subnotificación, como cambios en los tipos de violencia prevalentes y restricciones al acceso a servicios de apoyo y protección, lo que sugiere la necesidad de mejorar los sistemas de notificación y garantizar que las víctimas tengan acceso a los recursos necesarios, independientemente de las condiciones externas, como la pandemia.

Los resultados sobre la violencia doméstica contra mujeres en sus ciclos de vida en la ciudad de Maputo entre 2019 y 2022 pueden aplicarse en la práctica para orientar diversas intervenciones y cambios en las áreas de salud, asistencia social y políticas públicas.

Algunas acciones prácticas pueden implementarse para enfrentar el problema de la violencia doméstica^5^, como:

- Fortalecimiento de los servicios de salud y de notificación para mejorar la identificación, la atención y la notificación, incluyendo la capacitación de profesionales de la salud para identificar signos de violencia, aun cuando las víctimas no lo refieran directamente; el establecimiento de protocolos de tamizaje sistemático en todas las consultas de salud, ginecológicas, pediátricas y geriátricas; y el mejoramiento del registro de datos y de la notificación para garantizar que todos los casos identificados sean registrados y reportados a las autoridades;
- Desarrollo de redes de apoyo integradas que ofrezcan asistencia integral a las mujeres en situación de violencia, creando centros de acogida donde las personas en situación de violencia puedan buscar ayuda inmediata y obtener apoyo legal, psicológico y médico, que sean accesibles y funcionen las 24 horas del día;
- Programas de sensibilización y educación comunitaria, implementando acciones como: programas educativos para informar a las comunidades sobre los diferentes tipos de violencia (física, psicológica, financiera, malos tratos y sexual) y las maneras de denunciar, por medio de campañas en medios de comunicación, charlas en escuelas y centros comunitarios y programas de radio; fomentar el empoderamiento de las mujeres, especialmente las más vulnerables, a través de programas de educación, cualificación profesional y apoyo económico, pues las mujeres con mayor independencia tienen mayor capacidad de denunciar a los agresores y de salir de relaciones abusivas;
- Implementación de servicios de apoyo remoto para garantizar que las mujeres en situación de violencia tengan apoyo incluso en situaciones de restricción de movilidad, habilitando teléfonos de emergencia gratuitos o aplicaciones que permitan la denuncia anónima; y sitios web o aplicaciones donde las víctimas puedan acceder a información sobre cómo buscar ayuda, además de ofrecer soporte emocional a través de chats con profesionales de salud mental;
- Intervenciones enfocadas en franjas etarias específicas para responder a las necesidades de cada grupo, centrándose en programas de prevención de la violencia sexual y de los malos tratos, dotando a las escuelas de programas de educación sobre derechos, autocuidado y cómo denunciar; ofrecer programas que aborden la violencia física y psicológica, con foco en estrategias de resiliencia, apoyo emocional y económico; y desarrollar intervenciones específicas para el combate del abuso y los malos tratos a mujeres mayores, incluyendo campañas de sensibilización sobre abuso financiero y negligencia y la creación de servicios de apoyo especializado;
- Monitoreo continuo y políticas basadas en evidencia: los gobiernos locales y nacionales deben utilizar los datos de las notificaciones y las encuestas sobre violencia para monitorear tendencias y desarrollar políticas públicas basadas en evidencia que atiendan las realidades de la población femenina en diferentes contextos;
- Capacitación continua para profesionales de la salud y de la asistencia social que actúan directamente con víctimas, con capacitaciones periódicas sobre las mejores prácticas para abordar casos de violencia, técnicas de acogida y procedimientos legales fundamentales para mejorar la respuesta de los servicios de salud y sociales; y programas de formación que enfatizan la necesidad de tratar los casos de violencia de forma sensible y adaptada a diferentes contextos culturales, económicos y sociales.

Con base en los resultados presentados, se sugiere que futuras investigaciones aborden temas como:

- Barreras al registro de denuncias: investigar cómo las medidas de aislamiento durante la pandemia afectaron la capacidad de las mujeres para denunciar la violencia, considerando factores como la falta de acceso a dispositivos electrónicos y la baja familiaridad con tecnologías en poblaciones vulnerables; y evaluar cómo ampliar el acceso y la efectividad de canales de denuncia alternativos, como plataformas en línea o servicios telefónicos;
- Violencia contra mujeres mayores y cambio en los patrones de violencia: investigar las causas detrás del aumento de los casos de malos tratos en mujeres mayores y la disminución de los casos de violencia física y psicológica; y comprender los factores que conducen a la vulnerabilidad financiera y a los malos tratos;
- Violencia en niñas: analizar más detalladamente el aumento de los malos tratos y la variación de los casos de violencia sexual en niñas menores de 18 años, identificando medidas preventivas y de protección más eficaces para esa franja etaria;
- Determinantes sociales y culturales de la violencia: investigar los factores sociales, culturales y económicos que contribuyen a la violencia contra las mujeres en la ciudad de Maputo; y estudiar cómo la pobreza, el desempleo, las normas de género y la estructura familiar influyen en las dinámicas de la violencia, además de explorar cómo esas variables afectan a las mujeres en los diferentes ciclos de vida.

Las limitaciones del estudio incluyen aspectos relacionados con la calidad de los registros en Maputo, los cuales presentaron variaciones a lo largo de los cuatro años estudiados, lo que dificultó un análisis más preciso. Adicionalmente, los datos proporcionados fueron datos agregados de los siete distritos municipales de la ciudad de Maputo, lo que impidió el análisis territorial de las ocurrencias. La ausencia de información sobre el perfil demográfico de las víctimas y de los agresores restringió la comprensión de las dinámicas de los casos. Por último, las variaciones en la forma de agregación de los datos por franja etaria dificultaron la comparación directa entre los diferentes grupos etarios.

## Conclusión

El análisis de la violencia contra personas del sexo femenino en la ciudad de Maputo, Mozambique, entre 2019 y 2022, revela patrones importantes en términos de variaciones etarias, temporales y contextuales, como la pandemia. Las mujeres adultas fueron el grupo más impactado por la violencia a lo largo de los años, lo que refleja una elevada exposición a diferentes formas de violencia, como la física, la psicológica y la financiera. Las mujeres mayores también presentaron una tasa de violencia considerable, siendo más propensas a sufrir abusos que las niñas, con destaque para el aumento de los malos tratos en los años 2020 y 2022. Durante el período pandémico, se observó una reducción de 42% en las notificaciones de casos de violencia en relación con el período prepandémico. Esta disminución puede estar asociada al confinamiento social, a los cambios en las rutinas diarias y a la reducción en la capacidad de denuncia, además del impacto socioeconómico causado por la pandemia, que alteró las dinámicas familiares y sociales, dificultando la identificación y el reporte de los casos de violencia. Así, esta reducción debe analizarse con cautela, pues puede no reflejar una disminución real de la violencia, sino más bien una menor notificación de los casos debido a las restricciones y a la dificultad de acceso a los servicios de protección.

Aun habiendo reducción de los casos durante la pandemia, es fundamental monitorear el escenario pospandémico para entender si la tendencia de reducción se mantiene o si surgen nuevos factores que puedan aumentar la violencia; capacitar a profesionales de la salud, de la educación y de la asistencia social para identificar e intervenir en los casos de violencia; además de sensibilizar a la población sobre el problema; continuar con la recolección de datos y el monitoreo de los casos de violencia, como estrategia importante para identificar tendencias emergentes en el combate a la violencia de forma eficaz.

En suma, aunque los datos apuntan a una tendencia de reducción de la violencia contra mujeres en la Ciudad de Maputo, aún hay desafíos por enfrentar, especialmente en relación con los malos tratos y la violencia sexual contra niñas. El fortalecimiento de las redes de apoyo y la promoción de políticas de prevención, junto con la consolidación de sistemas de vigilancia y de protección social, son fundamentales para abordar este grave problema social y garantizar la seguridad y el bienestar de la población femenina, promoviendo un entorno más seguro y acogedor.

## Declaración de Disponibilidad de Datos:

Los conjuntos de datos relacionados con este artículo estarán disponibles previa solicitud al autor correspondiente.
